# Human iPSC-Based Modeling of Central Nerve System Disorders for Drug Discovery

**DOI:** 10.3390/ijms22031203

**Published:** 2021-01-26

**Authors:** Lu Qian, Julia TCW

**Affiliations:** 1 Nash Family Department of Neuroscience, Icahn School of Medicine at Mount Sinai, New York, NY 10029, USA; janeqian25@outlook.com; 2 Ronald Loeb Center for Alzheimer’s Disease, Icahn School of Medicine at Mount Sinai, New York, NY 10029, USA; 3 Department of Genetics and Genomic Sciences, Icahn School of Medicine at Mount Sinai, New York, NY 10029, USA

**Keywords:** induced pluripotent stem cells (iPSC), CNS disease modeling, drug discovery, toxicity screening, three-dimensional cerebral organoids, astrocytes, neurons, Alzheimer’s disease, COVID-19

## Abstract

A high-throughput drug screen identifies potentially promising therapeutics for clinical trials. However, limitations that persist in current disease modeling with limited physiological relevancy of human patients skew drug responses, hamper translation of clinical efficacy, and contribute to high clinical attritions. The emergence of induced pluripotent stem cell (iPSC) technology revolutionizes the paradigm of drug discovery. In particular, iPSC-based three-dimensional (3D) tissue engineering that appears as a promising vehicle of in vitro disease modeling provides more sophisticated tissue architectures and micro-environmental cues than a traditional two-dimensional (2D) culture. Here we discuss 3D based organoids/spheroids that construct the advanced modeling with evolved structural complexity, which propels drug discovery by exhibiting more human specific and diverse pathologies that are not perceived in 2D or animal models. We will then focus on various central nerve system (CNS) disease modeling using human iPSCs, leading to uncovering disease pathogenesis that guides the development of therapeutic strategies. Finally, we will address new opportunities of iPSC-assisted drug discovery with multi-disciplinary approaches from bioengineering to Omics technology. Despite technological challenges, iPSC-derived cytoarchitectures through interactions of diverse cell types mimic patients’ CNS and serve as a platform for therapeutic development and personalized precision medicine.

## 1. Introduction

Central neural system (CNS) diseases are perplexed with variable genetic, epigenetic and environmental factors [1]. Despite progresses in neuroscience, therapeutic development of most CNS diseases is challenged by difficulties in translating clinical efficacy. Statistically, less than 10% of potential CNS drugs are clinically approved compared to 15% for non-CNS drugs [2]. Pharmaceutical development of CNS disease therapies is thwarted with a high rate of clinical trial attritions, and an estimate of 8.1 years is needed for the Phase II and III development of CNS drugs, which is 2 years longer than that for non-CNS drugs. The fact that much clinical attrition occurs during the late phase of clinical trials [3] implicates insufficient bridging between pre-clinical discoveries and in-patient results.

In comparison with a two-dimensional (2D) induced pluripotent stem cell (iPSC) platform, a three-dimensional (3D) culture system characterizes disease pathologies with a more complex microenvironment. For example, a monolayer culture may be insufficient to demonstrate abnormal neural tubes folding in Spina bifida, but a 3D culture can comprehensively recapitulate the geometric folding and facilitate mechanistic studies on Spina bifida during early neural development [4]. Moreover, a 3D culture provides essential microenvironmental cues including intracellular interactions, cytoarchitectural organization and physical stresses necessary to underlie pathophysiological alterations in vivo [5].

In this review, we will overview a variety of iPSC-based cellular models and their impacts on revolutionizing the paradigm of drug discovery. First, we will review promising opportunities of human iPSC disease models over primary cell culture and animal models in pre-clinical drug development. Then, we will introduce up-to-dated 3D iPSC models integrated with multi-disciplinary techniques to dissect complex pathophysiological features of CNS diseases, their ground-breaking success in uncovering and recapitulating disease pathologies and a potential for further improvement. We will illustrate how diverse and cutting-edged iPSC-based disease modeling can help improve our understanding of CNS diseases and pioneer therapeutic development in the context of prevalent and rare neurological disorders. We will also cover application of iPSC-based models on facilitating high-throughput drug toxicity screening, and finally discuss current limitations of iPSCs and their technological extension for drug discovery in the foreseeable future.

## 2. Benefits of Using iPSC Models over Animal and Primary Cell Line Models

In vivo animal models have been frequently adopted in drug screening to predict clinical manifestations. For example, utilization of drosophila and zebrafish enables cost-effective high-throughput assays and rapid toxicity screens [6]. However, animal models face difficulty in precisely recapitulating human disease etiology and drug responses, because their genetic expressions and cellular metabolisms are intrinsically different from those of the human. For instance, repeated failures have been observed in translating therapeutic successes on amyotrophic lateral sclerosis (ALS) rodent models to ALS patients, which implicates inadequate animal simulation of human ALS etiology and raises concerns about using animals for ALS modeling and drug screening [7]. Transgenic mouse models in Alzheimer’s disease (AD) may provide another example. Familial AD is genetically driven, and typical molecular hallmarks of AD include pathological extracellular Aβ plagues and phosphorylated Tau tangles. However, due to species difference in the amino acid sequence of the amyloid precursor protein (APP), wild-type mouse models fail to develop Aβ plaques [8,9]. One solution is to overexpress mutant human *APP* gene. Tg2576 mice express human APP (APP695 isoform) with a double Swedish mutation (*APP*^K670N/M671L^) driven by the *PrP* promoter, resulting in significant overexpression of *APP* and plague formation in the frontal and temporal cortices, hippocampus, and cerebellum [10]. APP23 mice also carry double Swedish mutations but in an APP751 isoform driven by the *Thy1* promoter [11]. In comparison with Tg2576 mice, APP23 mice develop more pronounced cerebral amyloid angiopathy (CAA) with immediately formed compact plagues and localized neurodegeneration not observed in Tg2576 mice [12]. Such difference in the severity of AD pathologies implicates the impact of different promoters and APP isoforms on the variation of neuropathologies in AD transgenic mouse models [13]. In addition, pathological manifestations by AD transgenic models relies heavily on familial AD mutations and background mouse strains. Nevertheless, actual sporadic AD patients embody much more complex and distinct pathogenesis than transgenic mouse models, which hampers the translation of pre-clinical successes in these models.

Aside from animal models, primary human cell lines are an alternative platform to model human diseases, discover or repurpose drugs, and define accurate toxicology endpoints [6]. However, certain primary human cells are not easily accessible and scalable for expansion. For example, primary neurons in the adult CNS are post-mitotic, rarely tumorigenic, invasive to obtain [4] and hence inappropriate for large-scale studies. Similarly, primary coronary endothelial cells can only be extracted from coronary arteries through a highly invasive procedure [14]. Expansion of these cells is a bottleneck of long-term experiments due to limited divisions before the cells entering a proliferation arrest. As a result, a pooled cell population from multiple donors is often rather used, even if the model contains various genetic backgrounds and disease phenotypes of different individuals. This limitation may cause inconsistent experimental outcomes (subtle phenotypes) and hamper patient-specific modeling of human diseases [15].

Human iPSCs are genetically reprogrammed to embryonic-like cells by exogenous expression of pluripotency genes in terminally differentiated somatic cells [16]. Emerging appreciation of iPSCs’ utility transforms these cells into a powerful platform for drug discovery due to their strong self-renewal and pluripotency to convert into any cell types of interest in the human body with an unlimited quantity. These characteristics allow recapitulation of disease phenotypes in more pathologically related cell types with a sufficient supply. In addition, iPSCs reprogramed from patient tissues harbor patient-specific genetic backgrounds and therefore present a new opportunity for personalized drug design in precision medicine to minimize individual-specific adverse effects. Applications of iPSCs can be further extended in combination with genome editing technology like the CRISPR/Cas9 system, which facilitates gene manipulation in targeted cell types and enables high-resolution comparisons of endophenotypes across variable demographics. Therefore, a human iPSC-based disease model possess the following advantages: (1) Easy accessibility and scalability, (2) personalized medicine to minimizes adverse mortalities or non-responders in pharmacological treatments, and (3) better appreciation of human disease progression and etiology [17].

## 3. Platforms for Drug Discovery Using Human iPSC Disease Modeling

### 3.1. Advancement from 2D to 3D Modeling

In drug discovery, pre-clinical in vitro screening of drug efficacy and toxicity is highly critical. *In vitro* selection accurately narrows down the input compound candidates for animal studies and prevents wastes in time and resources [18]. In particular, regulators in the industry endorse a ‘fail-early’ principle to mitigate risks and seek continuous appraisals of technological advances to reduce early-stage costs associated with new drug development before clinical trials especially phase III, which is the most expensive phase of clinical trials [18,19]. Accordingly, several in vitro models have been designed to offer phenotypic screening during early drug development and allow visualization of cell morphological parameters as an endpoint to evaluate drug responses [18]. Traditional 2D cultures are preferred for their easy manipulation and control of culture environment but lack tissue-specific architectures and cell-matrix interactions. In contrast, 3D cultures provide an opportunity to recapitulate the essential effect of extracellular matrix (ECM) to regulate cellular behaviors and harbor physical factors such as ECM stiffness, oxygen tension, and temporal-spatial growth factor gradients reflective of the in vivo. Furthermore, additional dimensionality of 3D cultures improves the spatial organization of cell surface receptors engaging in intracellular interactions and exerts physical constraints on cells that lead to different cellular responses [19]. Another factor contributing to different drug sensitivity, migration and invasion, and cellular morphology of 2D and 3D cultures is the differential gene/protein expression due to an acquired cellular adaptation to different culture environments [18]. Specifically, cells in a monolayer culture are under a stretching stress caused by flattened substrates in an unnatural state, which may alter the expression of cancer genes promoting cell survival and skew the drug responses in vitro screening. These convoluted features enable 3D cultures to present cellular interactions, differentiation, mechano-responses, and microenvironments that are more physiologically relevant than a 2D culture model.

### 3.2. Development of Organoids to Model Diseases

Organoids are a type of 3D cell culture that resembles natural microenvironments from aspects of cell type diversity and intracellular communications, cell-matrix interactions, and convoluted nutrient transport systems [20]. Existing 3D cell cultures/aggregates can be generated mainly through two routes: (1) A scaffold-assisted structure with biomaterials or (2) a scaffold-free spheroid formed through self-assembly [13]. A scaffold/matrix-based 3D cell culture can be achieved by seeding cells on a 3D matrix or suspending cells in a liquid matrix followed by polymerization or solidification. Common scaffolds can be categorized into bio-derived scaffolds such as Matrigel and hyaluronic acid, and synthetic material-based scaffolds such as Polyethylene glycol (PEG), polyvinyl alcohol (PVA), and polycaprolactone (PLA) [19]. Scaffold-free 3D cultures are usually generated in suspension and have been frequently applied on generating cerebral organoids.

Cerebral organoids, also known as ‘mini brains’, reproduce fetal brain development and therefore serve as a powerful platform to study human development and CNS diseases. Self-organization during organoid differentiation generates complex and discrete anatomic regions resembling the human brain, including the dorsal cortex, ventral forebrain, retina, hippocampus, choroid plexus, and midbrain–hindbrain boundary [21,22]. Kadoshima et al. report forebrain-like structures in neural embryoid bodies (EB), in which basal progenitors in the outer subventricular zone of the self-formed 3D cortical neuroepithelium are similar to the outer radial glia found in the human brain [23]. Specifically, cortical organoids contain a large ventricular zone with SOX2-positive ventricular radial glial cells aligned in a polarized structure with the lumen and apical surfaces expressing adherent junction proteins [24]. Another organoid method is serum-free floating culture of embryoid body-like aggregates with quick reaggregation (SFEBq) [21]. In SFEBq, the dissociated human iPSCs aggregate in suspension, gradually form EBs, and differentiate to neuroepithelial rosettes that are heterogeneously polarized due to various rostral-caudal identities. Such heterogeneity can be manipulated through FGF signaling where the enhanced FGF signaling promotes rostralization of cortical progenitors, while the attenuated FGF signaling determines a caudal fate of the progenitors. Closely following the establishment of cerebral organoids, researchers generate more regionalized brain organoids such as hippocampus, pituitary, and hypothalamic organoids [25,26,27], which enable to form aggregates resembling different anatomical structures for mechanistic studies. For example, by fusing dorsal and ventral forebrain organoids, researchers establish a dorsal-ventral axis to reveal GABAergic interneuron migration and integration along the axis [28,29]. A low-shear stress has also played an increasingly important role in EB-to-organoid differentiation because the fluid dynamics in a spin-culture system not only promotes nutrient diffusion but also maximizes pluripotent stem cell propagation by keeping cell aggregates below the critical size of a diffusion limit [22,30]. Meanwhile, Pasca et al. have developed human cortical spheroids (hCSs), a cerebral organoid model that embodies gliogenesis and neurogenesis [31,32,33]. In particular, neurons in hCSs develop functional synapses through intimate interactions with surrounding non-reactive astrocytes. These hCS-neurons acquire miscellaneous anatomic structures in diverse cortical layers found in the human brain and carry transcriptional signatures resembling the developing human fetal brain. These cerebral organoids present neural division and migration patterns consistent with the human brain development, including inter-kinetic nuclear migration and can serve as an effective tool to reproduce human neural development in vivo [34].

### 3.3. Development of Blood-Brain-Barrier Organoids

One bottleneck in developing neurological therapeutics is the low efficiency of pharmacological drugs to cross the blood–brain-barrier (BBB) and reach targeted regions in the CNS. The BBB in vivo comprises brain endothelial cells that express molecular transporters and efflux pumps and are held by tight junctions and endothelial-interacting cells like pericytes and astrocytic end-feet [35]. Several approaches have been developed to improve drug delivery in the CNS. For instance, ligand-mimicking angiopeptides are generated to bind with the low-density lipoprotein receptor-related protein 1 (LRP1) to hijack the BBB transcytosis. Another method is to tag drug molecules with a cell-penetrating peptide that readily crosses the BBB [36,37]. However, only a few formulations of transporter-mediated transcytosis succeed in clinical trial transitions; significant challenges still remain in extrapolating animal study data to humans, including an indiscriminate uptake of transcytosis formulations that encompass major organs through the circulatory system [38,39,40], abnormal brain perfusion and degeneration of BBB structural components under pathological conditions [41]. Although algorithms have been generated to predict drug penetration in vivo, they are not fully reliable and require empirical confirmation [35]. Therefore, pre-clinical assurance of candidate compounds to penetrate the BBB is important to avoid attrition in clinical trials.

**Figure 1:**
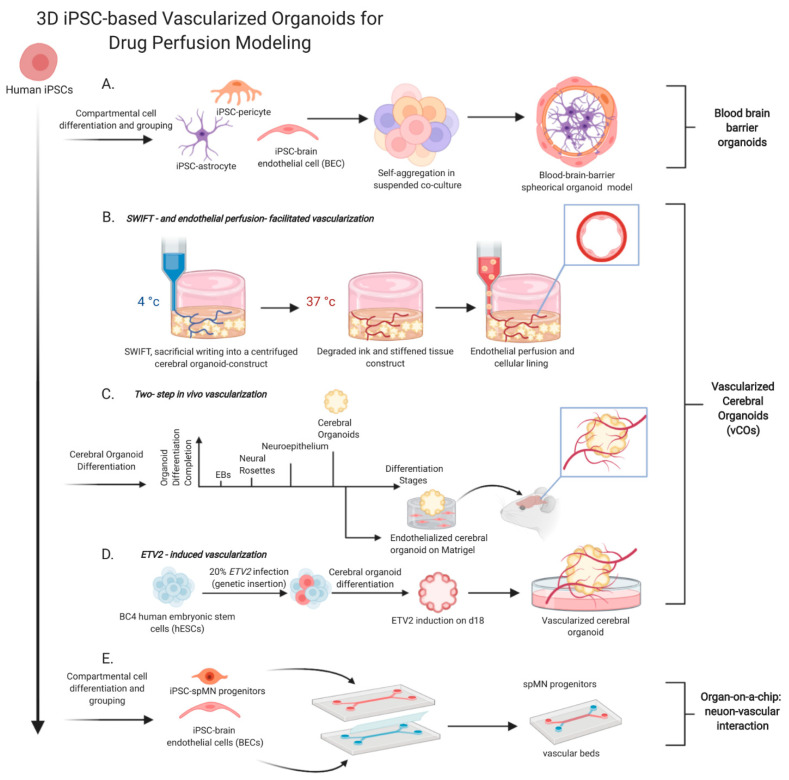
3D induced pluripotent stem cell (iPSC)-based vascularized organoids for drug perfusion modeling. (**A**) iPSC-derived blood-brain-barrier model. Astrocytes, pericytes and endothelial cells are generated from human iPSCs and mixed in suspension for self-aggregation. The resulted spheroid comprises an inner layer of astrocytes, intermediate layer of pericytes, and an outer layer of endothelial cells. (**B**) Scaffold-dependent vascularization of organoids through sacrificial writing into functional tissues (SWIFT). Cerebral organoids are mixed and compacted into a solid-like construct in an appropriate extracellular matrix medium. The sacrificial gelatin ink is 3D typed into the tissue construct. The tissue construct solidifies with increased temperature, while the sacrificial ink melts away, leaving tubular structures embedded within. Perfusion of endothelial cells endothelializes the interior of the channels and form vascular beds. (**C**) External revascularization of human iPSC-cerebral organoids within mouse brain cavity. Non-vascularized organoids are seeded onto endothelial containing matrigel droplets DIV 34 and transplanted onto raw edges of the resection cavity in the mouse brain. (**D**) *ETV2*-facilitated endogenous vascularization of cerebral organoids in vitro. A mixture of BC4 hECs and ETV2-infected BC4 hECs is prepared with a 20% cell infection rate. On day 18 of organoid differentiation, viral induction is initiated. Endothelial tubes are formed along the organoid differentiation and continuously evolve with increased structural complexity. (**E**) A dual-channeled microifluidic organ/organ-on-a chip (OoC) to model neural-vascular interactions. Human iPSC-derived spinal neural progenitors (hiPSC-spNPs) are seeded on the top channel and iPSC-derived brain endothelial cells (BECs) on the bottom. Vasculature develops as the neural progenitors differentiate into spinal neurons with mediated signaling between two different cell types.

Most of the existing in vitro BBB models are generated from primary animal brain endothelial cells (BECs), but species discrepancies in abundance and function of transporters may estrange these animal BBB models from the human BBB physiology [42]. Primary human BECs can be obtained through human brain biopsies but are still subjected to limited culture scalability, rapid loss of phenotypes in vitro, and suboptimal barrier properties. With advancements in iPSC applications, human iPSCs appear as a new source of BECs. For example, amniotic fluid cells (AFCs) have been used of iPSC reprogramming and BBB modeling. Because AFCs are derived from embryonic and extra-embryonic tissues, they share a closer origin to the embryonic stem cells [43]. Particularly, AFCs resemble characteristically the mesenchymal stem cells and harbor pluripotency to differentiate into a wide range of cell types [44]. Non-integrating episomal reprogramming is used to transform AFCs into iPSCs, which are further differentiated into BECs and form a BBB-like monolayer membrane in a two-compartment transwell system composing two layers with different cell types in one well [42]. However, a transwell-derived monolayer model is oversimplified with geometric irregularity that confers leakiness of the endothelial membrane [35]. A multicellular BBB-organoid model is therefore created to enhance the integrity of BBB-like endothelial structures. A mixture of BECs, pericytes and astrocytes is cultured in suspension and allows self-assembling into spheroids [35] (Figure 1A). These BBB-like spheroids are segmented into three ultrastructural compartments: BECs lining on the outer layer, pericytes in the intermediate layer with a direct contact with BECs, and astrocytes in the interior [45]. The BBB organoid surface forms an impervious barrier displaying essential BBB modulators, including tight/adherent junctions and P-glycoprotein efflux pumps to regulate molecular trafficking. A prevalent approach to evaluate small molecule transport across the BBB is matrix-assisted laser desorption/ionization mass spectrometry imaging (MALDI-MSI). MALDI-MSI has been used to assess the BBB-organoid integrity by measuring perfusion of BKM120 (high BBB penetrance) and dabrafenib (low BBB penetrance). MALDI-MSI on BBB-organoid sections after compound incubation reveals dramatic accumulation of BKM120 but an undetectable presence of dabrafenib. This BBB organoid model is also used to discover new brain-penetrating small peptides. Cell-penetrating peptides (CPPs) are peptides capable of crossing cell membranes. A small number of CPPs, such as HIV-1 Tat and SynB1, possess brain-penetrating capability. Cho et al. screen against a panel of 16 fluorescently tagged CPPs with BBB spheroids and compare the result with that of intravenous injection in mice models. The result shows that the in vitro BBB spheroid model demonstrates chemical permeability similar to that of in vivo models but needs more efforts to simulate the shear stress and blood flow as in the native BBB.

### 3.4. Development of Vascularized Brain Organoids

One limitation of many cerebral organoids is a lack of CNS vasculature. The complex vessel network interpenetrates the human brain during early neural development and provides a steady exchange of oxygen, nutrients, and inductive biochemical. The vasculature also serves as a structural template for early brain development and compartmentalization. The absence of vasculature in cerebral organoids restricts perfusion, causes necrosis compromising self-renewal and differentiation of progenitors, hinders the cortical plate formation and maturation of cerebral structures, and induces premature neural differentiation in the outermost layer [1,46,47]. Nonetheless, recent advancements in tissue modeling have succeeded in developing vascularized organoids to improve the perfusion and structural development.

Diverse strategies have been proposed to create vascularized organoids with enhanced oxygen perfusion. Existing vascularization approaches can be classified in two types: Biomaterial-assisted network fabrication and spontaneous vascularization. An example of scaffold-dependent vascularization is sacrificial writing into functional tissues (SWIFT) through embedded 3D printing (Figure 1B). Traditional bioprinting requires cellular and extracellular components in defined shapes and rapid crosslinking to develop a tubular construct [48,49]. To strengthen the structural support during network fabrication, Lewis and colleagues first demonstrate an embedded 3D printing by writing a viscoelastic and sacrificial ink within acellular hydrogel and silicone matrices [50,51]. Such fugitive bioink is later designed to be reversible in crosslinking/polymerization and can be degraded by temperature variations or appropriate chemical/enzymatic agents, leaving a robust tubular structure and functional integrity [48,52]. Integrating this advencement of bioprinting with iPSC-organoids, Skylar-Scott et al. introduce a sacrificial writing to organoid-matrices and generate organ-specific tissues with high density, maturation and desired functionality [53]. Cerebral organoids from large-scale microwell arrays are compacted to assemble a high-density SWIFT construct (~400,000 organoids per SWIFT construct) (Figure 1B). The cerebral organoids are mixed in appropriate extracellular matrix medium to form a slurry and solid-like living tissue matrix that displays viscoplastic characteristics at a low temperature. The sacrificial gelatin ink is injected in the SWIFT construct and distributed omni-directionally to form cylindrical filaments within the matrix. Subsequent temperature increase from 4 °C to 37 °C induces melting of the sacrificial gelatin ink and stiffening of the cerebral organoid matrix, which facilitates the removal of the ink and formation of embedded interconnected tubular channels. Perfusion of human umbilical vein endothelial cell through the channels endothelializes the luminal surface of bifurcating vascular networks. Interestingly, the sacrificial ink does not adversely affect the microarchitecture of nearby organoids within the SWIFT construct, which is evidenced by intact ventricular structures within individual cerebral organoids.

From a perspective of spontaneous vascularization, studies have focused on in vivo organoid transplant and endothelial co-culture [46]. For example, transplantation of human cerebral organoids in rodent brains increases the functionality, maturity, and diversity of phenotypic cells and bestows more complex and organized cytoarchitectures lacked in vitro [54]. Such improved physiological characteristics owe largely to steady distribution of oxygen and nutrients throughout the transplanted organoids due to local vascular integration as well as reception of host-produced paracrine signals that guide and endorse cell differentiation. Regarding co-culture-facilitated human-cell driven vascularization, Pham et al. report a strategy of external revascularization that penetrates into the core of the cerebral organoids during early development (Figure 1C) [55]. Specifically, non-vascularized organoids are seeded onto endothelial cells (ECs)-containing Matrigel droplets and allowed maturation in vitro. These EC-conditioned organoids are then transplanted onto raw edges of resection cavity in brains of immunodeficient mice for local vascular integration [55]. Transplanted EC-coated organoids present a robust ingrowth of human CD31 positive capillaries in a continuous and tubular structure that penetrates from the outermost layer into the core of the organoids. This strategy enables one to model developmental perineural plexus and recapitulate cerebral development with detailed cytoarchitecture of the whole-brain organoids. On the other hand, the fact that no human capillaries infiltrate the local mouse vasculature might be attributed to a lack of hypoxic angiogenesis, of which the process is controlled by the activation of hypoxia-inducible factor-1 (HIF-1) in response to oxygen deprivation [56]. Low HIF-1 activation suppresses the activation of downstream vascular endothelial growth factor A (VEGFA), a chemo-attractant that stimulates proliferation and migration of endothelial cells along the angiogenic gradient for hypoxic angiogenesis. Besides vascular integration, some studies bridge CNSs of mouse and human organisms by showing neural axon extension from human organoid grafts into the adult mouse brains, conversely mouse microglial colonization into the transplanted human graft [57].

Cakir et al. report endogenous vascularization in brain organoids through genetically inserting human *ETS* (E26 transformation-specific) variant 2 (*ETV2*) in pluripotent stem cells [58] (Figure 1D). E26 is an avian leukemia retrovirus [59], and *ETV2* is an *ETS* transcription factor that induces genes in VEGF and Notch signaling pathways involved in the determination of hematopoietic and endothelial cell lineages [60]. Mechanistically, *ETV2* regulates cardiovascular development and participates in pathophysiological angiogenesis as well as endothelial reprogramming from dermal fibroblasts [58,61]. Cakir et al. derive cerebral organoids from a mixture of BC4 human embryonic stem cells (hECs) and *ETV2*-infected BC4 hECs. While a small fraction (20%) of *ETV2* viral infection does not interfere with the development of cortical structures or transcriptional and morphological features of the vascularized cerebral organoids (vhCOs), *ETV2* promotes early formation of CD31 positive endothelial tubes that continuously evolve with increased structural complexity and robust expression of endothelial markers in the lumen of ventricular-like zones compared to the control. Fluorescein isothiocyanate (FITC)-dextran perfusion further demonstrates vascular perfusion within vhCOs, indicating development of functional vessel networks endorsed by *ETV2* expression. Meanwhile, the fact that vhCOs express more synapsin 1 and possess more electrophysiologically active neurons suggests significant contribution of vascular-like structures to neuronal maturation. Of note, the vhCOs exhibit essential characteristics of the in vivo BBBs: up-regulation of tight junction markers such as claudin-5 (CLDN5) and the presence of pericytes and astrocytes in the lumen of ventricular-like zones. In particular, trans-endothelial electrical resistance (TEER) analysis shows significantly elevated resistance of the vhCOs than the control COs but comparable to other reported 3D BBB models. As an exemplified AD model, treatment of Aβ_1-42_-oligo on vhCOs recapitulates neurotoxicity-induced malfunctioning of the BBB, which is evinced by an observation of FITC-dextran leakage due to damaged tight junctions. Therefore, the use of *ETV2*-reprogramed ECs produces vascularized cerebral organoids and provides a novel avenue to resolve hypoxia-induced necrosis without laborious epithelial coating or co-culture. Further, the vhCOs can accurately recapitulate the physiological features of the CNS, including neuron-epithelial interactions and BBB functions. Collectively, this strategy eases the process of vascularizing cerebral organoids, structurally and functionally imitates the human brain, and acts as a scalable platform for pathological investigation and drug development.

### 3.5. Limitations in Current Organoids Models

Recent advancements in cerebral organoid studies have expanded methodologies to generate BBB and vasculature for modeling human brain development and diseases. Cerebral cortical organoids have attracted much attention because the cerebral cortex is the most evolutionarily unique and expanded region in the human brain and frequently affected in diverse neurological disorders [24]. Transcriptomic profiling of the entire cerebral organoid and single cell RNA sequencing have demonstrated similarities between the cortical organoids and the human fetal brains for overlapping epigenetic signatures. However, despite this genetic relevance, studies on cerebral organoids are still in infancy and require improvement for disease modeling. Specifically, several limitations need to be addressed.

There are several technical issues associated with organoid generation. First, many cortical organoids display incomplete cortical folding [24]. Rapid cortical neurogenesis from the outer SVZ is a source of cortical surface expansion and gyrification (around gestational week 23 in pregnancy) during early brain development. However, cortical organoids are developmentally immature for gyrification. Consequently, these organoids lack the folding of the pia surface and plate layers underneath. Second, the self-organizing process in organoid generation is spontaneous without strict control, which leads to heterogeneity in spatial and proportional organization of individual cerebral organoids and inconsistency between experiments. Such developmental heterogeneity leads to batch effects, hinders reproducibility and interpretation of disease phenotypes and introduces noises that can obscure the true signal [1,62,63]. Last, most SFEBqs do not provide supportive tissues and body axes [64,65]. Although a cerebral organoid can develop discrete brain regions, it is difficult to arrange them into specific anatomical patterns identical to those of the human brain without any external axial patterning signals.

Moreover, whether cerebral organoids can capture aging-dependent phenotypes in neurodegenerative disease still remains questionable. Such concern is deepened with the fact that iPSC-derived organoids exhibit fetal or embryonic transcriptomic profiles that cannot be easily matured by extended culture in vitro [54,66]. Although excitatory and inhibitory neurons in cortical organoids are electrophysiologically functional with spontaneous burst-like firing activities and trains of action potentials upon current injection, neuron subtype-specific firing patterns and plasticity are still obscure. Additionally, neurons sampled from cortical organoids tend to present a heterogeneous degree of functionality and maturity due to continuous and non-synchronized neuronal differentiation in the organoids [24]. The fact that most of the differentiated organoids are confined to early-to-mid embryonic development evinces intrinsic immaturity of these models, making it difficult to reiterate phenotypes of an aged CNS.

Cerebral organoids generated from ectodermal development do not possess mesodermally originated immune cells like microglia that participate in neuronal development, migration and homeostasis in the human brain [67]. Microglia are the major immune cells that respond to pathological changes in the CNS microenvironments. Pathological activation of microglia results in heightened microglial phagocytosis and up-regulation of inflammation-related molecules. For example, microglia-associated neuroinflammation is a common AD phenotype involved in the disease initiation and progression. Studies have demonstrated that microglial Aβ phagocytosis triggers a release of IL-1β that elicits the secretion of NO and TNFα and aggravates amyloid plaque formation and neurodegeneration. In addition, the absence of microglia in cerebral organoids hinders microglial engagement in neural–glial interactions and limits recapitulation of microglia-associated neuroinflammation in AD, if no co-culture is given [1].

### 3.6. Development of Microfluific Chip to Model Diseases

Microfluidics emerges in response to an urged demand for miniaturized biomedical device, sensor innovation, and drug discovery. Application of microfluidics ranges from nanostructure synthesis and cell analysis to neural probe development in drug delivery [68]. A microfluidic device comprises a series of miniaturized components: Microchannels, microvalves, micropumps, micromixers, and microseparators. The fact that microfluidics requires only a small amount of fluid eases the process of sample preparation. Moreover, inclusion of highly flexible and elastic polymer materials like siloxane elastomers enables microfluidic devices to overcome a mechanical mismatch between rigid electronics and soft bio-tissues [69]. Specifically, flexible microfluidics installed in soft neural tubes helps maintain desired geometric deformation, electrical surges, and attributes of CNS tissues and avoids neuro-inflammation caused by heterogeneous device–tissue interfaces of traditional implants. Microfluidics also well-addresses concerns in drug discovery and preclinical tests that are time-consuming and costly. In particular, a microfluidic system helps elevate high-throughput screen capacity and result-reproducibility through automation of experimental pipelines; microfluidic cartridges pre-loaded with reagents simplifies assay procedures with a lower cost [70]. Therefore, incorporation of miniaturized microfluidics in drug development fosters the advancement in technological and financial efficiency of high-throughput screening.

Microfluidic organ/organ-on-a-chip (OoC) is engineered as a product of convergence between tissue engineering and biomedical micro-electric mechanical systems [70]. OoC simulates human pathophysiology with a variety of cell types and creates a biomimetic microenvironment that largely approximates human pathological conditions than a traditional monolayer-culture. In comparison with conventional organoid culture that presents heterogeneous and uncontrollable diffusion of exogenous morphogens inducing cell type differentiation and segregation [71,72], OoC is designed to contain microchannels as a sink of soluble factors to generate a stable gradient of morphogen diffusion across cell-hydrogel constructs [73]. OoC improves greatly the credibility of pre-clinical testing in vivo drug screening. Recent studies have integrated iPSCs with OoC to improve multi-tissue differentiation and potential of personalized medicine. Specifically, the iPSC-combined micro-engineering system emulates micro-volume cytoarchitecture in the CNS and enables co-culture of distinct cell types in vitro [74]. For example, comparative analysis of native spinal cord tissues and iPSC-spinal motor neurons in a 2D culture system shows that iPSC-spinal motor neurons do not actively express functional or mature markers beyond 4-6 gestational weeks or in pro-longed culture [75]. In contrast, an iPSC-spinal cord chip system increases effectively the spinal neuronal activity, enhances gene expression for neuronal differentiation, induces genes for vascular-neuronal interaction, and displays in vivo-like transcriptomic profiling [74]. In detail, a dual-channel OoC chip is designed for neural-vascular interaction and consists of human iPSC-derived spinal neural progenitors on the top channel and brain microvascular endothelial cells (BMECs) on the bottom channel (Figure 1E). Transcriptomic profiling reveals up-regulation of transcription factor *ISL1* in BMECs, implicating mediated signaling between the derived spinal motor neurons (spMNs) and vasculature. Such mediation is evinced indirectly by up-regulation of downstream angioneurins in the co-culture. Of note, expression of somatostatin (*SST*) in addition to other upregulated neuronal genes is detected in spMNs as its expression from GABAergic inhibitory interneurons serves as a critical regulator of motor cortex activity during motor learning in human brain and characterizes neocortex neurons with hyperactivity and a low threshold for spontaneous potential firing. Interestingly, hyperactivity of SST interneurons is an attribute to sustained inhibition of cortical motor neurons and cell deaths in an ALS mouse model [76]. Collectively, the iPSC-spinal cord OoC increases physiological relevance of an in vitro spMN model and improves its capacity to investigate human neurological pathologies.

However, several challenges exist in OoC to epitomize the human physiology. First, successful implementation of the chip model requires determination of an applicable size of each organ, perfusion conditions, proper surface-to-volume ratio for vascularization, and integration with feasible models to present the disease state [77,78,79]. Second, microstructures of channels in this chip may restrain cell growth and cause a formation of air bubbles within the tiny channels, leading to a challenge for long-term experiment designed to study developmental pathophysiology [80]. Third, an OoC model cannot circumvent the limited environmental reproducibility that also present in scaffold-based organoid models. Organogenesis often requires basement membrane extracts like hydrogel to support 3D structural development and morphogenesis. However, these membrane extracts usually consist of poorly standardized compositions and batch variability, which worsen the controllability of environmental factors [64,81]. Forth, little is known about the effect of polymers and fluid dynamics on cell behaviors and metabolite adsorption in a microfluidic construct, which may affect cellular function and humoral interaction between tissues [78]. Six, although recent studies have proposed multi-organ-on-a-chip as an advanced system to emulate organ-organ crosstalk, for example, long-term co-culture of liver spheroids with human neuronal tissues [82], the culture system lacks organ-specific conditions and microenvironmental cues because on-chip environments are usually identical across all organs/organoids and therefore may compromise the functionality and maturation of individual organs/ogranoids [83]. Last but not least, from the technical perspective labor training in specialized laboratory techniques is required to achieve prevalent application of OoCs [70]. Thus, simplicity and flexibility are desired to improve systematic operation and bench-top analysis of microfluidics.

## 4. Application of 2D and 3D iPSC Models for Drug Discovery

Despite progresses in genetics of neurological disorders, translation of genetic findings to mechanistic conclusions has been a challenge in human disease modeling [84]. Development of iPSC models well-addresses this situation, in which patient iPSC-derived phenotypic cells preserve and endogenously express disease-associated genetic variations and provide an access to study endophenotypes that bridge genetic profiles and cellular phenotypes [84,85]. Although the field has been moving forward to 3D models, 2D monolayer iPSC models, especially with patient iPSC-derived astrocytes and neurons, have been increasingly incorporated to recapitulate the human brain context of neurological disorders in vitro, associate molecular pathogenic mechanisms with various genetic risk factors, and provide a platform for drug discovery and therapeutic development. We will discuss how human iPSC modeling for drug discovery transforms the paradigm of neuropsychiatric research over the past few years from a perspective of several neurological diseases, including Alzheimer’s disease (AD), Parkinson’s disease (PD), amyotrophic lateral sclerosis (ALS), Huntington’s disease (HD), Rett syndrome, and Familial dysautonomia as well as virally induced CNS-related diseases such as ZIKA virus disease and Coronavirus disease 2019.

### 4.1. Alzheimer’s Disease (AD)

AD is the most common form of dementia resulting from neurodegenerative disease characterized by progressive neuron loss, cognitive declines, and memory loss. Non-cognitive symptoms like depression- and apathy-associated neuropsychiatric alterations may also arise as the disease progresses [86,87]. These miscellaneous manifestations increase the complexity of pathogenic mechanisms. Current knowledge about AD pathology comes extensively from studies on familial early-onset AD (fAD or EOAD) with defined and penetrant mutations in amyloid peptide precursor protein (*APP)*, presenilin 1 *(PS1),* and presenilin 2 *(PS2)* [88,89,90]. However, little is understood about the pathogenesis of sporadic late-onset AD (sAD or LOAD), which counts for the majority of AD diagnoses with unclear causal mutations. Genetic, environmental, and demographic risk factors are considered attributes to LOADs and complicate the disease etiology and mechanisms. Several biomarkers have been identified in AD, including senile plagues comprised of Aβ aggregates resulted from APP cleavage and intracellular neurofibrillary tangles of hyperphosphorylated tau protein [90]. Both Aβ oligomers and hyperphosphorylated tau are involved in neurotoxic processes. Although various therapeutic strategies targeting AD-related pathways have been tested on animal models, they have failed in clinical trials [1].

Over the past decades, AD research has confronted with challenges in determining LOAD pathogenesis and developing effective therapeutics to prevent or reverse AD relevant physiology. To investigate AD pathologies and facilitate the drug screening, iPSC-based neural models have been developed to imitate AD phenotypes in CNS. Both LOAD and EOAD iPSCs have been reported to successfully differentiate to cortical neurons and exhibit classic AD phenotypes such as Aβ accumulation, Aβ42/Aβ40 ratio dysregulation, and tau protein hyperphosphorylation as well as promoted reactive oxygen species (ROS) and DNA damages [90,91,92]. Kondo et al. have utilized AD iPSC-derived neurons as a platform for drug screening and found combined anti-Aβ cocktails beneficial in inhibiting Aβ plaque deposition in both EOAD and LOAD [93]. With this model, they have assessed the efficacy of docosahexaenoic acid (DHA) in improving AD neuron performance and revealed that DHA attenuates stress responses in AD neurons, whereas phenotypic variations of the drug efficacy present among patient samples [91]. Such phenotypic heterogeneity highlights the necessity to sub-categorize AD patients and neurons for effective compound selection in drug screening.

**Figure 2:**
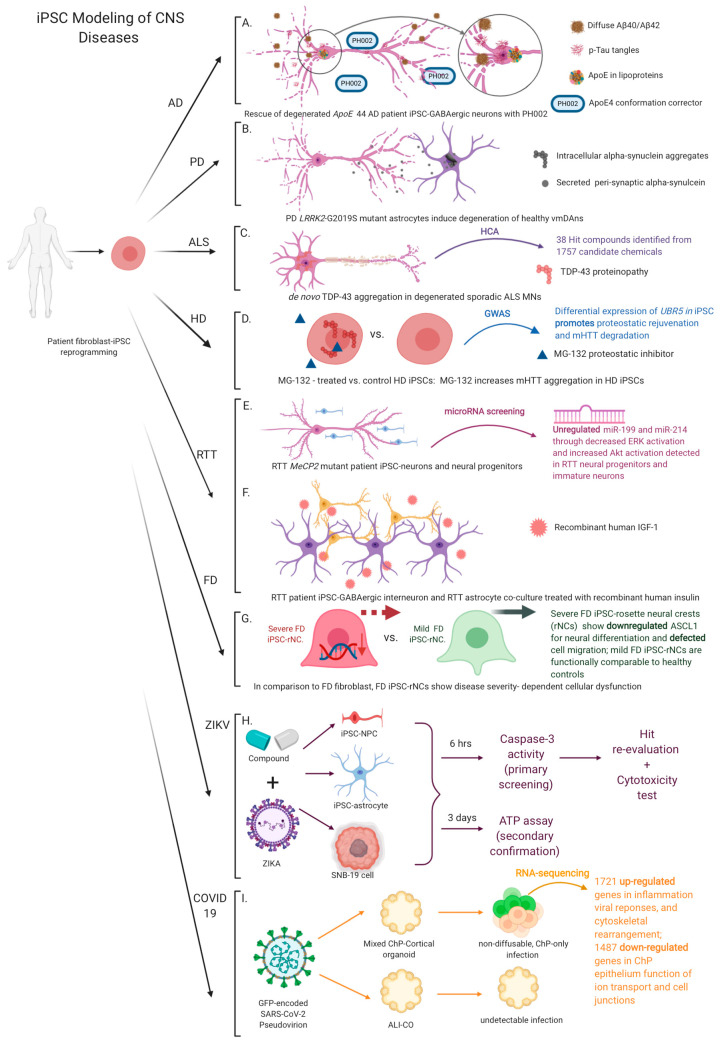
iPSC modeling of diverse CNS diseases for phenotype studies and drug discovery. (**A**) A proof-of-concept experiment to conformationally correct *APOE4* in Alzheimer’s disease (AD) with the compound PH002. PH002 improves cytopathologies of *APOE* 44 human iPSC-GABAergic neurons with reduced p-tau accumulation, Aβ40/Aβ42 production/secretion, and ApoE4 fragments. (**B**) A co-culture model of astrocytes and ventral midbrain dopaminergic neurons (vmDAns) derived from Parkinson’s disease (PD) patient-specific iPSCs carrying *LRRK2* G2019S mutation. Co-culture of *LRRK2* G2019S astrocytes with healthy vmDAns induces α-syn toxicity and vmDAns neuron degeneration as observed in PD patient brains. (**C**) Model of *de novo* TDP-43 proteinopathy and motor neuron (MN) degeneration in sporadic amyotrophic lateral sclerosis (ALS) patient iPSC-MNs. Automated robotics is applied on a high-content assay to screen against 1757 bioactive compounds. Based on the evaluation of reduced TDP-43 aggregation, 38 hit compounds are identified to effectively reduce the percent cells containing TDP-43 aggregates. (**D**) Utilize Huntington’s disease (HD) iPSCs as a model to study reprogramming-associated anti-mHTT aggregation compared to aggregate-free HD iPSCs. Treatment of the proteasomal inhibitor MG-132 increases mHTT aggregates in HD iPSCs, indicating a correlation between anti-mHTT aggregation and proteasomal homeostasis. Genome-wide association studies (GWAS) analysis shows upregulation of UBR5 particularly at the iPSC stage, which rejuvenates the proteasomal function of HD iPSCs to actively degrade mHTT aggregates. UBR5 thus serves as a potential therapeutic target to remove mHTT aggregation in HD patient cells. (**E**) Generation of neural progenitors and immature neurons from isogenic Rett syndrome (RTT) patient iPSCs to model *MeCP2* mutation-associated neurogenic deficiency during the early brain development. In comparison with the healthy control, *MeCP2* mutant neural progenitors and neurons show upregulated microRNA miR-199 and miR-214 in microRNA screening. Molecularly, immunoblots show decreased ERK activation and increased AKT activation, implicating a possibility of delayed neuron differentiation. (**F**) Treatment of recombinant human IGF-1 to improve neural performance in RTT. RTT iPSC-astrocytes are found to reduce the amount of terminal ends of RTT iPSC-GABAergic interneurons in co-culture. Short-term administration of IGF-1 modestly improves the neurite length of RTT-neurons in a diseased environment. (**G**) Model familial dysautonomia (FD) of various disease severities with FD iPSC-rosette neural crests (rNCs). In comparison to a healthy control, mild FD rNCs do not display differences in cell migration or the expression level of *ASCL1*, a proneural transcriptional factor for early neurogenesis and progenitor differentiation. In contrast, severe FD rNCs show compromised cell motility and decreased ASCL1 expression, indicating a disease severity-dependent functional defect that might shape the accuracy of disease modeling. (**H**) Compound screen flow chart for ZIKV therapy development. Three different cell types are used as a platform for drug screen: iPSC-neural progenitor, iPSC-astrocytes, and glioblastoma cell (SNB-19). SNB-19 and neural progenitors are exposed to ZIKV infection and screened against compounds that reduce apoptotic caspase-3 activity. The derived primary hits are re-evaluated on all cell types along with cytotoxicity assay. ATP assay is used as secondary confirmation of post-infection cell survival. (**I**) Utilize human iPSC-Choroid plexus (ChP) and cortical organoids to investigate neurotropism in SARS-CoV-2 infection. GFP-encoded pseudovirion is made to intracellularly visualize viral invasion. When cortical organoids mixed with ChP cells are exposed to the virus, only ChP cells but not neural progenitors or neurons show positive infection signals; infection does not spread to nearby cortical regions but infected ChP cells lead to global transcriptomic dysregulations in multiple cellular functions (top). When air–liquid-interface cerebral organoids (ALI-COs) are introduced with greater neuronal maturity to examine the viral infection in neurons, signal is undetectable relative to the positive control, evidencing a preferred attack on ChP cells and a lack of viral entry in neural cells (bottom).

Another example of iPSC-based modeling in AD is to study the gain of toxic apolipoprotein E4 (ApoE4) effects in human iPSC-neurons [94]. *APOE4* is identified as the major genetic risk factor associated with increased tau neurofibrillary tangles and Aβ aggregate deposition. Relative to its low-risk common isoform ApoE3, ApoE4 molecule is marked with a Cys-to-Arg-112 replacement, and homozygous *APOE4* brings about a 65% lifetime risk estimate to develop AD by age 85 [94,95]. Although post-mortem human brain tissues can serve as a study model, they only exhibit the end-stage disease characteristics and miss early pathologies along the disease progression. Another research hindrance is the species differences in animal models that lead to incomplete recapitulation of AD hallmarks. Such limitation is illustrated by human *APOE* knock-in (KI) mouse iPSC (miPSC)-neurons where human *APOE3*-KI and *APOE4*-KI miPSC-neurons exhibit no difference in the Aβ40 and Aβ42 deposition, implicating species-dependent ApoE regulation of Aβ processing [94]. To circumvent this innate phenotypic discrepancy, Wang et al. establish human iPSC lines from homozygous *APOE3* and *APOE4* patients and differentiate them to mature neurons [94]. Molecular analyses of these neurons show a higher ratio of intracellular ApoE fragments to full-length ApoE as well as elevated tau phosphorylation (p-tau) and Aβ secretion in *APOE* 44 neurons. Wang et al. categorize iPSC-neurons into GABAergic, glutamatergic, and dopaminergic neurons. Among these neuronal subtypes, only *APOE* 44 GABAergic neurons show neurodegeneration at a late stage of differentiation. Specifically, p-tau is not only accumulated in axons of human iPSC-GABAergic neurons and contributes to axonal degeneration, but is also mislocalized in the soma and dendrites of neurons, leading to dendritic deterioration. This observation suggests a high susceptibility of GABAergic neurons to ApoE4-induced neuronal degeneration. On the other hand, *APOE* knockout (KO) neurons show a similar level of p-tau and Aβ40/Aβ42 secretion to that of *APOE* 33 neurons. Yet, re-introduction of human *APOE* 44 to *APOE* KO neurons retrieves a high level of p-tau tangle accumulation, Aβ40/Aβ42 secretion, and GABAergic neuronal loss, demonstrating a gained toxic function of ApoE4. To examine whether these iPSC-neurons can serve as a model to develop AD therapies, Wang et al. design a proof of concept experiment by treating *APOE* 44 human iPSC-neurons with PH002, a small molecule structure corrector known to render ApoE4 molecule an ApoE3-like conformation (Figure 2A). As a result, PH002 significantly ameliorates p-tau accumulation, Aβ40/Aβ42 production/secretion, ApoE4 fragments and GABAergic neurodegeneration. Collectively, these findings confirm the effectiveness of ApoE conformational correction in rescuing AD phenotypes and contribute to guide and improve corresponding pharmaceutical strategies to treat AD.

Down syndrome (DS) iPSCs have been used as an alternative to model AD phenotypes such that DS iPSC-derived neurons express similar AD cytopathies, early amyloid aggregates and overexpressed tau that is hyperphosphorylated and mislocalized into the linear foci in soma and dendrites [96,97]. These overlapping cytopathies make DS iPSC-derived neural cells a flexible platform for AD drug screening. However, it is important to remind that DS and AD have different disease pathogenesis and genetic attributions. The ambiguous disease characteristics may raise concerns about the effectiveness of symptomatic treatments using compounds derived from phenotype-reliant drug screening, which overlooks AD-specific mechanisms. Moreover, monolayer cultures of a single cell type are limited in recapitulating wholesome abnormalities of the AD brain that comprises convoluted cytoarchitectures and interactions among multiple cell types responsible for synaptic pruning, non-cell autonomous glial signaling in CNS inflammation and so on.

To overcome limitations in 2D modeling, 3D iPSC-based neural models are generated as a tool for drug discovery and mechanistic studies. A recent study by Park et al. incorporates a microfluidic chamber to develop a human AD tri-culture that consists of neurons, astrocytes, and microglia [98]. In this microfluidic model, the central chamber is loaded with a 3D co-culture of iPSC-neurons and astrocytes that interact with adult microglia on the angular chamber. This model enables to recapitulate active microglial recruitment, neurotoxicity-induced axonal cleavage, and NO release, which are detrimental factors for neurons and astrocytes in AD. This tri-culture system facilitates the understanding of AD neural-glial interactions and opens an opportunity to create a more physiologically relevant platform for drug development. Cerebral organoids have been developed to successfully reproduce critical AD cytopathies, including progressive accumulation of Aβ aggregates, amyloid plaque deposition, and neurofibrillary tangles [99]. Choi et al. have implemented AD brain organoids to evaluate the pharmacological responses of candidate compounds targeting various AD phenotypes [100]. Cerebral organoids derived from AD patient iPSCs harboring *APP* and *PS1* mutations display robust extracellular Aβ deposition and tau hyperphosphorylation in both the soma and neuritis. By disrupting Aβ generation with β- or γ-secretase inhibitors, Aβ accumulation is reduced along with improved Tau pathology. This result implicates that the familial AD-Tau pathology might result from excessive Aβ accumulation. Administration of glycogen synthase kinase 3 (GSK3) also evidences the regulation of tau phosphorylation mediated by an Aβ pathway. Raja et al. have utilized self-assembled brain organoids derived from familial AD patient iPSCs to model AD pathologies [88]. During the AD organoid differentiation, Raja et al. observe the formation of neuroectoderm that expresses neuronal MAP2 and possesses SOX2-positive neural progenitors. This neuroectoderm differentiates into a rosette-like rolling structure that finally becomes volumetrically developed organoids at a late stage of differentiation. When the conditioned medium is examined, Aβ secretion is higher in familial AD organoids than healthy controls. Time-dependent elevation of the amount and size of Aβ aggregates is also detected in familial AD organoids accompanied with progressively increased deposition of Aβ oligomers. Subsequent Tau pathology appears in familial AD organoids and sprawls out to the majority of the surface area of organoids. In addition to AD molecular hallmarks, familial AD brain organoids display sizeable endosomes and trend to produce larger EAA-1 positive endosomal particles than healthy controls. It is noteworthy that this familial AD brain organoid model unveils an endosomal pathology not yet reported in mouse models and reproduces disease pathologies comprehensively reflective of human patients, thus expanding an opportunity to study AD mechanisms.

Another inspiring implementation of human iPSC-derived cerebral organoids is to investigate the role of BACE2 as a gene-dose dependent AD suppressor [101]. DS patients who bear chromosome 21 trisomy (T21) are more susceptible to develop AD due to an extra copy of *APP* gene on the third chromosome 21 with an increased risk of AD dementia. However, unlike single *APP* triplication (Dup*APP*) resulting in 100% AD penetrance, T21-driven AD onset is observed in only 70% DS patients by age of 60. Such different odds of disease implicate potential modulation of AD onset by additional genes on the third chromosome 21. β-secretase 1 (BACE1) is the main β-secretase responsible for APP cleavage on chromosome 21, and β-secretase inhibition has been an attractive approach to reduce Aβ peptide production, while *BACE2*, a homologue juxtaposed to *BACE1*, remains less understood. Some roles of BACE2 in APP processing have been identified as: a pro-amyloidogenic β-secretase, a θ-secretase to prevent Aβ formation, and an Aβ-degrading protease (AβDP) in an extremely acidic environment. Particularly, θ-secretase cleaves the APP β-CTF fragment between the amino acid 19 and 20 and produces an Aβ1-19 fragment; AβDP degrades efficiently Aβ40/42 and yields Aβ1-20 or 1-34. Interestingly, SNPs in *BACE2* gene are associated with the onset age of dementia among DS patients [102], and a de novo intronic deletion within a single *BACE2* allele has been reported to cause EOAD in a 50-year-old euploid patient [103]. It is therefore hypothesized that *BACE2* overdose contributes to modify the AD onset in DS patients with T21 [101].

To recapitulate increased soluble Aβ observed in DS forebrains and explore the impact of *BACE2* on Aβ cleavage, Alic et al. reprogram iPSCs from primary keratinocytes of the same DS patient mosaic for T21 and normal disomy 21 cells and create isogenic T21 and disomic 21 (D21) iPSC lines [101]. They also generate Dup*APP* iPSC lines from a euploid familial early-onset AD (FEOAD) patient with an extra copy of *APP* but normal disometric *BACE2*. Self-assembled cerebral organoids are derived from each iPSCs. Immunoprecipitation-combined with mass-spectrometry and ELISA are used to evaluate Aβ cleavage products in conditioned medium. The results show significantly increased ratios of Aβ1-34 and Aβ1-20 to amyloidogenic peptides with unaltered amounts of Aβ α-site cleavage products in T21 organoids. Such preference of BACE2-mediated APP processing is however not perceived in D21 or Dup*APP* organoids. The data suggest that the increased β-secretase activities and Aβ40/42 clearance may stem from the third copy of *BACE2*, and a single third copy of *APP* is insufficient to promote AβDP-like activities found in T21. Furthermore, immunostaining of Aβ1-40, Aβ1-34, and BACE2 in organoid sections shows a stronger signal in neurons of T21 organoids than that of isogenic D21 organoids, evidencing a correlation between increased AβDP activities and T21 BACE2. Strong co-localization of Aβx-34 (AβDP product) with BACE2 staining is also detected in acidic lysosome sub-sets that express LAMP2 around neutritic plaques. In contrast, co-localization of Aβx-34 with BACE1 in lysosome or with LC3A in Aβ-containing macro-autophagic vacuoles is insignificant. Importantly, closely following LAMP2 co-localization, Aβx-34 is found co-localize highly with HSC70 that translocates proteins to the lysosome lumen for degradation after being detected by LAMP2 on the lysosomal membrane [101,104]. Co-localization with HSC70 suggests an involvement of chaperone-mediated autophagy (CMA) in the degradation of amyloidogenic Aβ peptides [101]. On the other hand, by CRISPR-knocking out a single BACE2 copy in T21 iPSCs and maintaining the rest of chromosome 21 trisomy, Alic et al. are able to generate BACE2-corrected trisomy organoids that show a decline in the amounts of putative BACE2-AβDP products and total BACE2-related non-amyloidogenic peptides. Such reduction suggests a gene-dose-dependent effect of *BACE2*. In comparison with parental iPSC-organoids that do not display any amyloid deposits on 100 DIV, those CRISPR-edited organoids fast develop AD-typical plaques in the cortical region by early 48 DIV and continue to progress with massive cell deaths. However, these pathologies can be reversed by a combinatorial treatment of θ-secretase inhibitor IV and γ-secretase inhibitor XII, leading to fewer neurons expressing pathologically conformed Tau.

Although pharmaceutical inhibition of BACE1 remains a strategy for AD treatment, it may cross-inhibit BACE2 function. To investigate the extent of BACE2 inhibition caused by BACE1 inhibitors, Alic et al. develop a new in vitro FRET assay to measure BACE2-driven AβDP activities [101]. Three BACE1 inhibitors are tested in the presence of recombinant human BACE2: LY2886721, β-Secretase Inhibitor IV, and γ-Secretase Inhibitor (DAPT). Of note, LY2886721 is the first BACE inhibitor to reach Phase 2 clinical trials in AD after the discontinuance of clinical development of LY2811376 [105]. The FRET assay shows that LY2886721 and β-Secretase Inhibitor IV suppress BACE2 cleavage in a dosage-dependent manner [101]. This finding provides an explanation to some clinical failures on BACE1 compounds and an insight to modify AD pharmacological treatments with fewer side effects.

### 4.2. Parkinson’s Disease (PD)

PD is the second most prevalent neurodegenerative disease than AD and affects over 6 million people worldwide predominantly above the age 65 [106,107]. Clinical features of PD include motor symptoms ranging from rigidity and resting tremor to bradykinesia and postural instability. Motor symptoms stem from a progressive loss of ventral midbrain dopaminergic neurons (vmDAns) in the substantia nigra pars compacta, and ~50% degeneration of dopamine neurons occurs in the midbrain at the onset of motor symptoms. Sporadic PD accounts for the majority of cases (~85%), while only a minority of PD cases are familial (~15%) [108]. Leucine-rich repeat kinase 2 (LRRK2) contains both a GTPase domain and a protein kinase domain and is involved in cellular autophagy. In PD pathogenesis, mutant LRRK2 binds LAMP2A, a receptor for CMA to degrade α-synuclein (α-syn), leading to functional inhibition of CMA complexes and to accumulation of toxic intracellular α-syn aggregates in vmDAns [107,109]. PD patient iPSC-derived neurons harboring *LRRK2* mutation have been reported to show impaired axonal outgrowth and deficient autophagic vacuole clearance [107]. Particularly, *LRRK2*-mutant iPSC-dopaminergic (DA) neurons show an altered CMA process that may contribute to abnormal α-syn accumulation and precede morphological manifests of neurodegeneration.

While most of the studies focus on vmDAn degeneration, emerging appreciation of astrocytic dysfunction provides a different angle to understand the PD pathogenesis in the human brain. Astrocytes under a normal condition uptake peri-synaptic α-syn through neuron-astrocyte interactions. Overexpression of mutant *SNCA* (α-synuclein gene) in astrocytes is known to compromise astrocytic integrity with the blood–brain-barrier and glutamate homeostasis and cause degeneration of vmDAns [110,111,112]. Similarly, *LRRK2* mutation not only affects DA neurons but also astrocytes. di Domenico et al. utilize patient-specific iPSCs with *LRRK2* G2019S mutation and differentiate them to functional astrocytes and vmDAns [107]. Co-culture of PD iPSC-astrocytes with healthy iPSC-vmDAns induces DA neuron degeneration as observed in PD patient brains (Figure 2B), while healthy astrocytes are able to partially rescue the diseased phenotypes of *LRRK2*-PD iPSC-vmDAns. Intracellularly, healthy iPSC-astrocytes transfected with *LRRK2* exhibit cytoplasmic accumulation of α-syn that is absent in control astrocytes. Co-culture of healthy iPSC-vmDAns with the transfected astrocytes induces α-syn accumulation and pathological morphology of neurons, including fewer and shorter neuritis and decreased survival of iPSC-vmDAns due to α-syn toxicity. On the other hand, CRISPR-corrected *LRRK2* G2019S astrocytes no longer accumulate intracellular α-syn and appear effective in preventing α-syn accumulation in control vmDAns. These data implicate that *LRRK2* mutation engages in disrupting vmDAn homeostasis. To determine whether enhanced lysosomal activity can eliminate astrocytic α-syn accumulation, a CMA activator (CA) (a chemically designed retinoid derivative) is treated on PD astrocytes to inhibit the retinoic acid receptor-α (RARα) signaling pathway and promote CMA functions for macroautophagy [107,113]. Interestingly, the treated cells show a significantly reduced level of α-syn and recovered perinuclear distribution of LAMP2A-positive lysosomes, approaching the state of healthy astrocytes [107]. Of note, treatment of CA facilitates the clearance of α-syn accumulation in control vmDAns conditioned with PD astrocytes and is able to alleviate the progression of neurodegeneration and partially restore neuronal survival.

In summary, implementation of patient iPSC-derived vmDAns and astrocytes reveals an insight in PD astrocyte dysfunction and identifies pathogenic α-syn accumulation an underlying attribute to neurocytotoxicity of DA neurons. Meanwhile, the PD iPSC-astrocytes serve as a platform to examine the feasibility of enhancing CMA and lysosomal activities to ameliorate PD-associated toxic α-syn accumulation. Accordingly, a class of CMA-promoting proteins could be used as a hit compound in high-throughput screening for PD drug development.

### 4.3. Amyotrophic Lateral Sclerosis (ALS)

ALS is an adult-onset, complex genetic neurodegenerative disease featured by muscle wasting and a progressive loss of upper and lower motor neurons [114,115]. ALS has two subtypes: Familial ALS (fALS) and sporadic ALS (sALS). Although sALS accounts for ~90% of ALS diagnoses, its genetic etiology still remains unclear [114]. In addition, growing appreciation of disturbed higher cognitive function in ALS patients has linked ALS and FTD [115]. Particularly, 15% frontotemporal dementia (FTD) patients develop ALS features and 51% ALS patients display aberrant neuropsychological manifestations indicative of frontal lobe dysfunctions [116,117]. Genetic evidence has indicated mutations in the same genes between fALS and FTD-ALS and supports the idea that FTD and ALS are a spectrum disorder [118]. One major mutation that causes fALS and FTD-ALS is *TDP-43* (TARDBP or TAR-DNA-binding protein-43), which is also found in sALS patients [114,115,119]. TDP-43 is a ubiquitous nuclear protein that binds to the TAR DNA sequence in the HIV-1 genome to regulate viral gene expression [120]. TDP-43 proteinopathy is a key neuropathological feature in the CNS of ALS patients and frontotemporal lobar degeneration (FTLD) patients and appears in forms of cytoplasmic-mislocalization, hyperphosphorylation, intranuclear, and cytoplasmic aggregates; a loss of nuclear TDP-43 leads to altered transcriptomic profiles and chromatin decondensation [114,119]. TDP-43 pathology is also found in Lewy body dementia and AD, implicating its role in mediating neurodegeneration. Given that none of the rodent models can recapitulate human TDP-43 proteinoapthy, it becomes valuable to gain insights of ALS pathomechanism and drug discovery from sALS patient iPSC-derived neurons carrying *de novo* TDP-43 pathology [114].

Burkhardt et al. reprogram iPSCs from skin fibroblasts of healthy individuals and sALS patients and differentiate these iPSCs to motor neurons (MNs) expressing MN identity hallmarks, ISLET1 and HB9 [114]. The iPSC-MNs are capable of electrical activities within two weeks of co-culture with human primary astrocytes and display spontaneous calcium transients that can be inhibited by Tetrodoxin treatment. Comparative studies of TDP-43 expression in different cell types show that MNs have a higher level of TDP-43 than other cell types like neural progenitors. Contrary to healthy iPSC-MN controls, TDP-43 molecules in sALS MNs appear to aggregate spontaneously without external stimulation or genetic inducers such as mutations in *TDP-43*, *GRN,* or hexanucleotide repeats in *c9ORF72* (Figure 2C). These sALS iPSC-MNs possess TDP-43-positive round structures morphologically similar to intranuclear inclusions reported in ALS and FTLD postmortem CNS. In addition, TDP-43 aggregates are preferentially detected in ISLET1/HB9-positive cells. To determine whether TDP-43 aggregates in iPSC-MNs are post-translationally modified, ubiquitin and phosphoserine 409/410-specific TDP-43 antibodies are used. The immunofluorescence shows only phosphorylation-mediated modifications of TDP-43 aggregates, which are similar to cytoplasmic TDP-43 aggregates found in the postmortem CNS of ALS patients. The data suggest that phosphorylation may precede uibiquitination in TDP-43 pathology.

To investigate the mechanism of TDP-43 aggregation, a high-content assay is developed. Burkhardt et al. utilize an automated robotics to screen against 1757 bioactive compounds on sALS patient iPSC-MNs [114]. By assessing immunofluorescent co-localization of ISLET1 and TDP-43 to determine the presence of aggregates in iPSC-MNs, 38 hit compounds are identified to effectively reduce the number of aggregate-containing cells in the primary screening. In the secondary screening, these selected hits are tested on iPSC-cortical neurons (upper MN-like cells) from the same sALS patient. Four categories of compounds are found to reduce aggregate-containing percent neurons in a dose-dependent manner: Cyclin-dependent kinase inhibitors, c-Jun N-terminal kinase inhibitors (JNK), Triptolide, and FDA-approved cardiac glycosides (e.g., Digoxin, Lanatoside C and Proscillaridin A).

In addition, iPSCs-derived MNs are used to investigate the effect of *TDP-43* mutation on cell autonomous or non-cell autonomous mechanisms that trigger MN dysfunction in fALS [115]. Wild-type TDP-43 proteins under a normal condition form cytoplasmic mRNP granules and facilitate bidirectional and microtubule-dependent granule transport [121]. In contrast, *TDP-43^M337V^* mutation in iPSC-derived MNs induces genetic dysregulation related to RNA metabolism and cytoskeleton functions [122]. In particular, *TDP-43^M337V^* iPSC-MNs show compromised expression of neurofilament-medium (NEFM) and neurofilament-light (NEFL) chains and impaired NEFL mRNP granule transport in axons. *TDP-43^A90V^* iPSC-derived neurons also show increased vulnerability to stress-induced cytotoxicity and reduced microRNA-9 expression [123]. Collectively, iPSC-derived ALS MNs bridge cellular models and human patient pathology with essential phenotypes consistent with the postmortem ALS data and provide a versatile tool to facilitate mechanistic studies and therapeutic development targeting TDP-43 proteinopathy.

### 4.4. Huntington’s Disease (HD)

HD is an autosomal dominant neurodegenerative disease featured by excessive CAG trinucleotide expansions in the exon 1 of huntingtin gene (*HTT*) [124]. Excessive CAG repeats translate into unstable polyglutamine (polyQ) expansions within the huntingtin protein and form insoluble, aberrant intracellular aggregates resulted from proteolytic cleavage [124,125], leading to cytotoxicity-induced neurodegeneration [126]. However, detailed mechanisms still remain elusive and require further investigation with a sophisticated HD model. A recent discovery of toxic oligomeric intermediates during mutant HTT (mHTT) aggregation updates the traditional ‘neurotoxic PolyQ aggregates’ paradigm and proposes an early-stage protective role of mHTT aggregates to sequester toxic oligomers [127]. Nevertheless, no decisive conclusions have been drawn regarding the HD pathogenesis.

HD patient iPSCs do not show compromised pluripotency or abnormal karyotype [128] and interestingly do not accumulate polyQ-expanded HTT aggregates [129,130]. Along the differentiation trajectory, both HD iPSC-derived neural progenitor cells and neurons develop disturbed gene expressions and show excitotoxic vulnerability and compromised survival. However, HD iPSC-neurons do not readily develop polyQ aggregates as the native HD neurons do. Such discrepancy from primary neurons indicates association between reprogramming-resulted proteostatic rejuvenation and reduced mHTT aggregation in HD iPSC-neurons, hinting on potential proteostatic rejuvenation to suppress mHTT aggregates in HD patients [131]. HD iPSC-models are therefore adopted as a phenotypic model to investigate the underlying mechanism of anti-mHTT aggregation and proteostatic rejuvenatiton for the development of HD treatment.

By blocking the proteasomal activity with a compound inhibitor MG-132, HD iPSCs appear to produce more HTT proteins and accumulate more mutant aggregates [131] (Figure 2D). To address whether such aggregate accumulation stems directly from proteasomal blockage without interfering other pathways, Koyuncu et al. inhibit global autophagy processes in HD iPSCs and measure the mHTT aggregates [131]. As a result, they detect a much lower level of mHTT aggregation and less compact packing of mHTT proteins with global autophagy inhibition than native aggregates found in HD patients or with proteasomal inhibition. This discrepancy suggests proteasomal dysfunction to be a more immediate attribute to HD mHTT aggregation. Through genome-wide association studies (GWAS), *UBR5* is identified as the gene significantly associated with HD mHTT aggregation. UBR5 is an E3 ligase associated with Lys48-linked ubiquitination and triggers proteasomal degradation [132]. Regardless of the diagnosis status, *UBR5* is transcriptionally upregulated in iPSCs and downregulated in neural cells [131]. This transcriptomic pattern explains a differentiation-dependent decline in proteasomal activities in HD iPSC-neural cells. On the other hand, ectopic over-expression of *UBR5* has been found sufficient to reduce the extent of HTT aggregation. UBR5 activity enhancement thus emerges as a therapeutic strategy to reduce mHTT aggregates and correct abnormal HD proteasomal activities.

### 4.5. Rett Syndrome (RTT)

RTT is a severe form of autism spectrum disorders (ASDs) featured by *de novo* mutations in the gene of X chromosome-linked gene methyl CpG binding protein 2 (*MeCP2*) [133]. MeCP2 is a multi-functional epigenetic regulator associated with diverse neuropsychiatric disorders and expressed on a time course related to neuronal maturation and synaptogenesis [134]. Interestingly, restoring MeCP2 function can halt neural circuit atrophy in mouse models even at a late stage of RTT and repair the disease manifestations with additional maturation. However, the effect of MeCP2 deficiency on human neurogenesis and neuron differentiation is not fully investigated in a monolayer or 3D culture.

To address this challenge, Mellios et al. generate neural progenitors and three-week immature neurons from isogenic RTT patient-derived iPSCs to mimic an early stage of human neural development and examine the MeCP2-assoicated neuropathology [135]. MicroRNA (miRNAs) screening shows robust upregulation of miR-199 and miR-214 in RTT neural progenitors and neurons relative to their isogenic controls (Figure 2E). Comparative analysis evaluating the expression of primary miRNA precursors and mature miR-199 and miR-214 indicates that such upregulation originates not from miRNA precursor transcription but from disturbed miRNA processing. A *MeCP2* knockdown neuronal model is created by introducing *MeCP2* shRNA (shMeCP2) to wild-type neural progenitors and differentiating to early neurons. This shMeCP2 model demonstrates an association between MeCP2 deficiency and miR-199 and miR-214 overexpression. In silico studies on potential targets of miR-199 and miR-214 reveal enrichment in MAPK (ERK/AKT) process. ERK and AKT are two significant factors involved in the embryonic brain development, in which ERK promotes neuronal differentiation, and AKT enhances the survival and proliferation of neural progenitors. Immunoblots on shMeCP2 neural progenitors reveal a decreased ratio of phosphorylation-activated ERK1/2 to total ERK1/2 and an increased ratio of phosphorylated AKT to total AKT. However, no significant changes in ERK and AKT activation are further detected after three weeks of neuron differentiation. Coinciding with diminished dendritic complexity and neuronal marker expression, these in vitro results reveal aberrant neurogenesis in prenatal RTT brains.

The iPSC-RTT model facilitates not only studies on pathophysiology but also serves as a platform for drug discovery. Studies have shown that overexpression of brain-derived neurotrophic factor (BDNF), a downstream target of MeCP2 signaling, helps improve RTT symptoms [136]. However, peripheral-intravenously administered BDNF does not easily cross the BBB in the CNS and requires conjugation with a BBB transport vector to facilitate transcytosis [137]. Alternative to BDNF, insulin-like growth factor 1 (IGF1) is a crucial activator downstream of MeCP2 signaling and can readily cross the BBB [134]. Therefore, IGF1 becomes a therapeutic candidate for RTT drug development.

Multiple models have been used to evaluate the effect of IGF1 on rescuing phenotypes of RTT. Administration of recombinant human IGF1 has been found to improve the lifespan, locomotor activities, heart rate, respiratory patterns, and behavioral deficits of *MeCP2* knockout mice [138]. Given these promising data in mouse models, Williams et al. proceed to human models by creating isogenic pairs of wild-type and disease iPSCs from RTT patients [139]. iPSC-astrocyte progenitors are differentiated from neurospheres and matured into astrocytes. While no developmental defects are detected during the astrocyte differentiation, primary mouse hippocampus neurons co-cultured with RTT astrocytes show significant shrinkage of neurites and soma sizes. Primary mouse neurons cultured in conditioned medium from RTT astrocytes also show similar morphological alterations. These results evince an adverse non-cell autonomous effect of RTT astrocytes. To examine whether these adverse neuronal effects could be replicated in human neurons, human GABAergic interneurons are generated from isogenic iPSCs and co-cultured with iPSC-astrocytes. Morphological analyses reveal that RTT astrocytes compromise human neurite development and reduce the amount of neuronal terminal ends regardless the disease status of neurons, whereas the wild-type astrocytes are able to improve the morphology of RTT interneurons. Moreover, short-term administration of IGF1 can modestly improve the total neurite length of human GABAergic neurons in pure RTT co-culture (Figure 2F), demonstrating the potential benefit of IGF1 to improve neuronal performance of RTT patients. Collectively, this study reveals a non-cell autonomous mechanism of RTT astrocytes as well as therapeutic effects of IGF1 in improving human RTT neural phenotypes, which may underlie a future direction in the RTT drug development.

### 4.6. Familial Dysautonomia (FD)

FD is a congenital neurodegenerative disease caused by a point mutation in IkB kinase complex-associated protein (*IKBKAP),* which results in variable and tissue-specific skipping of exon 20 in the *IKBKAP* gene and reduces the yield of the functional IKAP protein, a scaffold protein involved in neurogenesis, neuronal survival, and peripheral tissue innervation [140]. FD symptoms include developmental delays and prolonged breath holding in children and autonomic and sensory nervous problems in adults (Genetics Home Reference, 2020). Typical clinical manifestation of FD includes a loss of afferent baroreceptor signaling that relays arterial pressure to the nucleus ambiguus and dorsal motor nucleus in the brainstem to regulate blood pressure [140,141]. Degeneration of these nuclei in FD therefore leads to blood pressure dysregulation and hypertensive vomiting attacks [140].

Human lymphoblast and fibroblast cell lines have served as a common platform for FD drug screening against bioreactive compounds. FD fibroblast cell lines are prevalently used of exploring FD pathological mechanisms regarding disrupted mitochondrial function [142], neurotrophic factor secretion [143], cell motility [144], and RNA splicing [145]. Similarly, FD lymphoblasts have been used in drug screening. In particular, among 1040 FDA approved compounds screened on human FD lymphoblasts, kinetin emerges as a therapeutic compound to effectively undermine mutant *IKBKAP* transcripts and concomitantly improve the wild-type *IKBKAP* expression in a dosage-dependent manner [146,147]. In vivo chronic treatment with kinetin has also been found to enhance neurogenesis and expression of peripheral neuron markers [4].

Given that the peripheral neural system (PNS) stems from the trunk neural crest and is severely affected in FD and that abnormal neural crest migration in brain development induces FD [148], neural crests appear more physiologically relevant for FD modeling than fibroblasts or lymphoblasts. Of note, FD patient iPSC-derived neural crest precursors are able to proliferate steadily after initial isolation and retain differentiation capacity after multiple passages. More importantly, these cells exhibit FD splicing defects and cytopathology. While primary FD fibroblasts do not show any differences that discriminate the disease severity, rosette neural crests (rNCs) display functional disparities between the mild and severe FD [149]. Specifically, severe FD patient iPSC-derived rNCs show defected cell migration and deficient expression of *ASCL1*, a proneural transcription factor that acts early on neurogenesis, regulates progenitor proliferation and induces differentiation in the CNS [150]. In contrast, mild FD patient iPSC-rNCs show migration and *ASCL1* expression comparable to that of the healthy control (Figure 2G). Accordingly, FD iPSC-neural crest modeling provides an opportunity to more accurately reproduce tissue-specific splicing and neurogenic defects and predict pathologies of peripheral sensory neurons [146,148].

Based on a correlation between reduced functional IKAP and increased disease severity, researchers have uncovered secondary mutations exclusively found in severely affected FD patients by utilizing whole-exome sequencing [148]. These mutations may act as genetic modifiers that exaggerate the disease severity. Such findings encourage the incorporation of FD iPSC-neural crest precursors in high-throughput screening and emulate a step-forward to pursue iPSC-based drug discovery. To integrate iPSC-neural crests with existing assays for FD bioactive compound screening, Lee and Studer design a screening trial against multiple compound candidates including Vitamin E derivative tocotrienols and polyphenol epigallocatechin gallate on traditional FD-fibroblasts followed by FD-iPSC neural crests with kinetin as a positive control [146]. However, only kinetin shows a robust rescue of splicing phenotype and improves the autonomous neuron generation, whereas the other compounds fail the first fibroblast screening and are excluded from the subsequent assays. It is possible that some of these compounds are overlooked and might show promising therapeutic potentials, if they are tested directly on a FD iPSC-neural crest model.

### 4.7. ZIKA Virus Disease and Coronavirus Disease 2019 (COVID-19)

ZIKA virus (ZIKV) is a mosquito-borne flavivirus that can be transmitted through mosquitos, sexual contact, and mother to fetus [151,152,153] and is known to cause congenital defects like microcephaly [154]. Various 3D cellular models have recapitulated ZIKV-induced microcephaly and defected development of the human cortex [133] and shown reduced thickness of neural progenitor cells (NPCs) and neuronal layers in early human brain development [155,156,157,158], Studies on ZIKV human forebrain organoids have proved selective viral attacks on neural progenitors that delay cell cycle progression, force premature neural differentiation and increase cell deaths [158,159]. Specifically, ZIKV infection of human organoids upregulates the expression of innate immune receptor Toll-Like-Receptor 3 (TLR3), and compounds competitively inhibiting TLR3 can improve ZIKV pathologies [158]. Pathway analysis has also shown an association between TLR3 activation and 41 genes involved in neuronal development, suggesting ZIKV-mediated disruption in neurogenesis. These cytopathologies induce a volumetric shrinkage of oragnoids, which resembles microcephaly observed in ZIKV patients.

Despite preclinical advancements in vaccine development, ZIKV prevention still remains unsolved [160,161]. Drug repurposing emerges as a new approach for ZIKV drug development. According to previous findings on ZIKV-induced NPCs attrition, Xu et al. design a compound screen utilizing caspase-3 activity and cell viability assay to identify potential therapies to resist effects of ZIKV infection [162]. The primary screening measures caspase-3 activity of human iPSC-NPCs, iPSC-astrocytes and glioblastoma SNB-19 cells after ZIKV exposure (Figure 2H). ATP content assay is used as secondary confirmation of cell survival after the viral exposure. By applying this screening campaign against essential bioactive compound libraries, 173 primary hits are identified to be effective in rescuing SNB-19 cells and iPSC-NPCs. Cytotoxicity re-evaluation of these compounds on all three cell types helps identify chemicals that reduce virally induced caspase activation and apoptosis (Figure 2H). In particular, Emricasan, a pan-caspase inhibitor, emerges as the most potent anti-apoptotic compound that eliminates detrimental effects on NPCs in ZIKV-infected brain organoids. Moreover, by examining the level of ZIKV protein NS1, ZIKV RNA, and ZIKV particles, Niclosamide (an FDA approved category B anthelmintic drug) and PHA-690509 (a cyclin-dependent kinase inhibitor) are identified as effective compounds to inhibit ZIKV infection at a post-entry stage, likely during viral RNA replication. These findings provide a tool to propel the development of anti-ZIKV therapies and resolve similar flaviviral diseases.

Another example of viral diseases is coronavirus disease 2019 (COVID-19), a global pandemic caused by the novel severe acute respiratory syndrome coronavirus 2 (SARS-CoV-2) and has resulted in over 96 million infection cases and more than 2 million deaths across 221 countries as of January 19, 2021 (https://www.worldometers.info/coronavirus). SARS-CoV-2 mediates its host cell entry through a spike (S) glycoprotein that comprises 2 subunits [163,164] where subunit S1 facilitates viral attachment to the angiotensin-converting enzyme 2 (ACE2), a host cell surface receptor, and subunit 2 enables virion-host membrane fusion [165,166]. Besides predominant respiratory syndromes, neurological complications appear in severe clinical cases and include seizures, confusions, psychosis, meningitis, and acute hemorrhagic necrotizing encephalopathy [167,168,169]. Importantly, SARS-CoV-2 RNA has been found in the cerebrospinal fluid (CSF) of COVID-19 patients with concomitant neurological symptoms [168] and patient brain autopsies [170]. However, it is unclear whether these symptoms result from neural infection, post-infection immune diseases, or sequalae of systemic diseases [171,172].

Both the BBB and the blood–CSF-barrier (B-CSF-B) are known to prevent blood-borne pathogens from invading the CNS. Unlike BBB that are structurally insulated by forming tight junctions between endothelial cells, pericytes, and astroglial endfeet, B-CSF-B consist of single-layered choroid plexus (ChP) epithelium to separate CSF from fenestrated stroma capillaries [173,174,175]. Given that B-CSF-B secretes pro-inflammatory cytokines into the CSF and is in close physical proximity to the stroma, an infiltration site of immune cells [176], the ChP epithelium becomes a target to study pathogenic invasion and elevated immune responses in COVID-19.

Current studies on SARS-CoV-2 infection and related drug efficacy mostly utilize human cancer cell lines [166,177,178,179] and transgenic animals that overexpress human *ACE2* [180]. However, cell line models are limited in recapitulating pathologies of diverse cell types in multiple affected organs and are insufficient to reiterate tissue-specific differential expression of ACE2 and susceptibility to SARS-CoV-2 infection. In addition, P53 mutations in human cancer cell lines affect SARS-CoV-2 replication and might fail to predict normal cellular responses to viral infection [181]. To address this limitation, Pellegrini et al. develop human iPSC-derived ChP and cerebral organoids to faithfully reproduce neuronal function and organization in vitro [182]. Single-cell RNA sequencing of ChP and telencephalic organoids reveals clustered expression of ACE2 and co-entry factors in ChP cells rather than neurons or neural progenitors. Immunostaining of ACE2 and a co-entry factor TMPRSS2 also shows strong signals in the ChP epithelium compared to the cortex. Intriguingly, subclustering analysis of ChP cells shows the highest expression of ACE2 and co-entry factors in lipid-expressing or mature ChP cells, suggesting a correlation between viral infectivity and ChP maturity. To examine neurotropism, Pellegrini et al. develop mature cortical organoids mixed with ChP [182]. GFP-encoded SARS-CoV-2 Spike pseudovirions are created to study spike-mediated infection (Figure 2I). Only ChP epithelial cells show 13% infection, and no infection is detected in the cortical region. To address a lack of infection in the cortical region, an air-liquid-interphase-cerebral organoid (ALI-CO) model that lacks ChPs but offers improved neuronal maturity is incubated with SARS-CoV-2 pseudovirions. However, no GFP signals are detected within ALI-CO neurons in comparison with the positive control (vesicular stomatitis virus G protein pseudotyped lentivirus), implicating a lack of viral entry. Thus, the model confirm that only ChP cells shows positive infection, but viral spreading to adjacent cortical neuronal regions is extremely limited (Figure 2I). On the other hand, though ACE2 expression is less prominent on the basal side of ChP cells facing the stroma, fluid-filled cysts show abundant viral invasion from the basal side, suggesting the necessity of ChP epithelium-vascular interactions to allow blood-borne pathogen infection. Specifically, disruptions of tight junctions in the ChP epithelium have been observed in early post-infection accompanied by anatomical alterations of ChP organoids, in which a leakage of organoid-originated CSF-like fluid is detected with the presence of CSF component APOJ in the surrounding media.

SARS-CoV-2 infection in ChP cells results in global transcriptional dysregulation. By performing RNA-sequencing of infected ChP organoids, Jacob et al. detect 1721 upregulated genes and 1487 downregulated genes [183]. Gene ontology analysis reveals that the upregulated genes are enriched in processes of viral responses, cytokine responses, cytoskeletal rearrangements and cell deaths, including elevated expression of inflammatory cytokines 7 (CCL7), inerleukin-32 (IL-32), CCL2, and IL-18. The downregulated genes are enriched in processes of ion transport, transmembrane transport, and cell junctions. Affected ion channels and transporters include AQP1, AQP4, and SLC22A8 that are important in maintaining CSF secretory function of ChP cells, indicating damaged ChP function. Interestingly, an increased level of phospho-p38 signaling due to viral infection is also observed in nearby uninfected cells, suggesting a substantial non-cell autonomous effect of SARS-CoV-2 infection.

Given no established prophylactic strategies or approved antiviral drugs for COVID-19 treatment, repurposing FDA-approved therapies might be an emergent approach to fast screen existing drugs for a new disease treatment. Human iPSC-ChP organoids may therefore serve as a model to not only investigate the disease mechanism but also identify epithelium-targeted, anti-viral drugs that could prevent or reduce SARS-CoV-2 induced neurological symptoms.

## 5. Application of iPSC Models for Drug Toxicity Screening

Preclinical toxicity screening of compound candidates is an essential step in drug development to examine the adverse effect and end point events during compound treatments. Side effects of in-patient therapies may include carcinogenesis and cardiotoxicity but particularly direct to cellular toxicity for CNS treatment. Rather than immediately transit from in vitro assays to patient trials, drug candidates undergo pre-clinical toxicity screening to compute a No Observed Adverse Effect Level (NOAEL) dosage used for clinical trials [184]. Toxicity screening is traditionally performed in vitro cell lines or in vivo animals, but the observed response is usually species-, organ-, or dose-dependent. Such discrepancies result largely from different enzyme-generated metabolites. Specifically, bioactivation is a metabolic process where reactive species are generated and lead to genotoxicity, covalent adducts, reactive oxidation species, and idiosyncratic drug reactions [185]. Animal models are not reliable in predicting reactive metabolites because their metabolic processes are naturally estranged from the human physiology.

High content analysis (HCA) and high-throughput screening (HTS) have become a preferred strategy for toxicity screening in drug development. Many of these approaches incorporate fluorescent probes in cellular sites to monitor multiple toxicity endpoints and phenotypic changes measured by multi-color visualization [185]. Novel modifications have further extended HTS applications, which include biologically compatible nanomaterials like zinc oxide nanowires to monitor microsome metabolism as well as electrochemical impedance spectroscopy to assess the drug toxicity by detecting morphological alterations of HeLa cells or fibroblasts in a 3D-hydrogel flow array. The emergence of human iPSC technology enables rapid HTS screening of thousands of drugs directly on multiple physiological relevant cell types in a large scale, helps circumvent interspecies dilemmas in disease modeling, and enables personalized HCA or HTS assisted-toxicity assessments with patient-specificity.

In neurodegenerative diseases, astrocytes appear as a target of pharmacological therapeutics for their pivotal roles in the CNS to promote regeneration of damaged neurons and protect the existing neurons from degeneration [186]. iPSC-derived astrocytes can therefore serve as a model to screen compound libraries against neurotoxicity. In a proof-of-concept experiment, Pei et al. characterize the feasibility of iPSC-astrocytes in toxicological testing by establishing a cytotoxicity screen panel against 80 neurotoxins and environmental compounds [187]. This screening examines cytotoxic responses of multiple different cell types including iPSCs, neural stem cells, iPSC-astrocytes, and iPSC-neurons. A viability assay, MTT [3-(4,5-dimethylthiazol-2-yl)-2,5-diphenyltetrazolium bromide], is used to measure cell proliferation and drug-induced cytotoxicity, in which MTT is reduced to purple formazan by mitochondrial dehydrogenase in viable cells and yields a colorimetric readout. Result of MTT assays shows cell type-dependent cytotoxicity with astrocytes displaying the greatest resilience to drug responses, and 41 out of 80 compounds are toxic to astrocytes. Such observed cytotoxicity is reproducible across genders and different genetic backgrounds of multiple individuals. Vice versa, iPSC-astrocyte models can be used of positively selecting protective therapeutics against harmful reactive metabolites. Oxidative damage is largely involved in the pathogenesis of many neurological diseases and is one of the pharmacological targets to halt the progression of CNS diseases. Phenotypic assays have been performed on iPSC-astrocytes in a high-density 536-well plate conditioned with hydrogen peroxide to screen against oxidation-protective compounds [186]. Morphological alterations are monitored to assess astrocytic responses to cytotoxicity. As a result, 22 chemicals from approximately 4100 candidate compounds elicit protective nuclear profiles in astrocytes, providing a beneficial perspective for therapeutic development that focuses on astrocyte protection.

Co-culture of iPSC-neurons and iPSC-astrocytes has served as a model to predict convulsion or muscular contractions induced by antiepileptic drug toxicity in nonclinical studies [188]. Convulsion is a clinical manifestation of seizures triggered by multiple different mechanisms that complicate disease diagnosis. The most frequent cause of convulsion is disrupted equilibrium between neuronal excitation and inhibition [189]. Although convulsions manifest CNS-related drug toxicity in clinical trials, little is known about the underlying CNS activities caused by convulsion-resulting antiepileptic drugs (AEDs) [188]. To determine the adverse effects of new AEDs and investigate the complex mechanism of convulsion, Odawara et al. measure electrophysiological activities of local neural networks on iPSC-cortical neurons by multi-electrode arrays (MEA) and evaluate the potential of drug-induced convulsion [188]. Neurons treated with Pentylenetetrazol (PTZ) and 4-Aminopyridine (4-AP) that are a GABA_A_ antagonist and a K^+^ channel antagonist, respectively, induce seizures through different routes. MEA measurements reveal that both 4-AP and PTZ increase the number of synchronized burst firings (SBFs) and epileptiform activities of the neurons. However, the burst histogram during a SBF shows that 4-AP induces peaks earlier at the beginning of SBFs and increases peak firings per time in comparison with PTZ. The MEA readouts also indicate that 4-AP increases significantly neuron SBFs in a dosage-dependent manner, which keeps rising even after the administration of phenytoin known to suppress SBFs. This observation is opposite to that of PTZ where the number of neuronal SBFs falls right after the phenytoin administration. To prove that the in vitro neuron-astrocyte co-culture model can predict reliably the authentic human response, Odawara et al. compare the in vitro frequency of local field potential (LFP) with human brain electrocorticogram (ECoG). Comparative analysis below LFP 250 Hz shows that iPSC-derived co-culture successfully recapitulates the enhanced γ and β wave components in human ECoG at a convulsive state. Collectively, this iPSC-neural network model appears effective in predicting toxicity-induced convulsion in the CNS and discerning action mechanisms triggered by different AEDs.

A more advanced in vitro model for drug toxicity testing other than a monolayer co-culture is a 3D-cytoarchitectural culture that embodies physiological and microenvironmental complexity. Sirenko et al. report recently drug toxicology evaluation by applying HTS on commercially available 3D human iPSC neural spheroids, composing cortical glutamatergic and GABAergic neurons as well as astrocytes [190]. A trial screen panel containing 10 neuroactive agents and 22 neurotoxins is established to validate the reliability of the 3D spheroid model to accurately detect neurotoxicity. A calcium oscillation assay to detect cytotoxicity-induced intracellular calcium disturbance/seizurogenic incidents in iPSC-neurons and MEA to evaluate electrophysiological dynamics of local neurons are used during screening against a panel of 87 chemicals representative of environmental hazards and neurotoxicity, covering typical neurotoxins such as pesticides, brominated and organophosphorous flame retardants. These assays find that 57% of candidate compounds are neurotoxic. Concentration-response profiling ranks these toxic compounds according to their effective concentration of neurotoxicity. Such ranking is then applied on associating in vitro toxicity with in vivo human responses to establish risk evaluation of these chemical compounds in industry use. This study reports a promising opportunity to commercialize cerebral spheroid/organoid and increase their accessibility for research and exemplifies the use of iPSC models in endeavoring toxicity screening for drug development and censuring chemical products that may impose potential environmental and health hazards.

## 6. Limitations of iPSC-Based Models for CNS Drug Discovery

Emergence of iPSC-based disease models revolutionizes drug discovery by providing unlimited patient-specific, pathologically relevant, and diverse human cell types. Genome-edited iPSC models allow flexible manipulation of disease phenotypes, facilitate pathogenic studies, and help identify disease targets for effective de novo drug designs. Nonetheless, researchers have come to acknowledge multiple challenges of using iPSC models.

As mentioned earlier in LOAD modeling, human iPSCs are not omnipotent to model all neurological diseases. In particular, they are limited in recapitulating aging-related neurodegeneration. Human iPSC-neurons have been reported to exhibit phenotypes that resemble fetal neurons, as evident that only a small number of neuron expresses mature synaptic markers during differentiation [191]. This is because the epigenetics of patient somatic cells is restored back to the embryonic stage during iPSC-reprogramming [6,192,193]. Particularly, age-associated markers in nuclear organization, heterochromatin, DNA damages, and ROS are erased during the process of iPSC rejuvenation [192] where the telomeres are elongated, and DNA methylation patterns are reset [194]. Aging or expanded cell culture is usually epigenetically marked with DNA methylation. A progressive loss of DNA methylation underlies replicative senescence [195], in which widespread hypomethylation and focal hypermethylation are perceived in cells marching towards replicative senescence demonstrated by whole-genome-nucleotide bisulfite sequencing [196]. Although senescence-associated DNA methylation can be reacquired through iPSC re-differentiation, the epigenetic age still remains relatively rejuvenated and only accelerates slowly upon differentiation [197]. On the other hand, the epigenetic reset may not be completed in iPSCs. Studies have found that iPSCs more or less sustain epigenetic memories from the tissue of origin in terms of residual DNA methylation [20].

Approaches have been proposed to enhance iPSC maturation and re-gain aging properties. Progerin overexpression is one example to mimic aging in iPSCs. Aberrant accumulation of progerin has been found to disrupt the lamin-A scaffold and indirectly contribute to the premature aging in Hutchinson-Gilford progeria syndrome. However, this approach introduces a new genetic variable and complicates the phenotype and interpretation of pathogenesis [6,198]. Similarly, extended in vitro culture cannot eliminate fetal properties, due to a lack of essential in vivo biophysical cues required for the human brain maturation. Moreover, in vitro cell culture will eventual lose the differentiation potential and exhibit altered cellular morphology, metabolic dysfunction, increased p53/p21CIP1 signaling, and senescence-associated activities [199].

To circumvent the dilemma of lacked aging-associated epigenetic features in iPSCs, ‘transdifferentition’ is proposed as an alternative to differentiate the desired cell types directly from somatic cells without intermediate rejuvenation. Ambasudhan et al. have reported a combinatorial use of miRNA (miR-124) and transcriptional factors (BRN*2* and MYT1L) to reprogram adult human fibroblasts into glutamatergic neurons that display typical neuronal morphology, marker expression, electrophysiological activities, and synaptic networks [200]. Mertens et al. have also optimized *Ascl1/Ngn2* based- and small molecule-enhanced neuronal transdifferentiation from human primary fibroblasts [201]. By comparing cellular features across age groups and cell types, Mertens et al. find that neurons derived from middle-aged donors show a loss of nucleocytoplasmic compartmentalization (NCC) comparable to that in equivalently aged fibroblasts. Age-dependent NCC impairment thus appears as an important functional indicator of human neurons in aging individuals. However, challenges still remain in this method in that the resources of primary somatic cells and the resultant cells are limited due to the lack of self-replicating/pluripotency and that an intermediate stage of neuronal induction is unstable due to epigenetic barriers in the source cells [202].

In addition, though protocols for pluripotency induction and downstream differentiation have been evolved, they are not completely standardized and under-development. Inconsistency in cell origins, transcriptional combination, subtype uniformity, and cell type purification methods causes heterogeneous phenotypes and ambivalent test results that may raise concerns about the validity of using iPSC-based models to predict authentic cellular responses in vivo [203,204].

Meanwhile, iPSC-based platforms still remain expensive for drug discovery. Individualized collection of donor cells and subsequent iPSC-reprogramming and differentiation are labor-intensive and highly time-consuming. For example, titrating for each donor requires multiple iterations and therefore complicates and elongates the process of disease modeling and makes HTS more difficult to achieve [205]. Moreover, enormous costs in generating and validating iPSCs remain a hurdle to expand iPSC application. Specifically, iPSC generation requires multiple validation including genetic fidelity and stability, potency determination, pluripotency marker expression, and post-thawed viability [206]. Thus, human iPSC banking is required to efficiently provide high-quality iPSCs to research facilities. However, most banks are non-profit and government owned, and only a limited number of iPSC banks exist worldwide [207]. Therefore, difficulties still exist in establishing a large patient cohort for disease studies to generate reliable clinical translation and statistically reduce the impact of genetic and clonal variables.

## 7. Future Aspects of iPSC-Based Models for CNS Drug Discovery

Although iPSC-based large-scale drug discovery is still in its infancy and requires efforts to optimize practicability, iPSC is undoubtedly a promising strategy to resolve long-lasting technical issues in disease modeling. Multi-disciplinary efforts are under development to enhance the technological feasibility of iPSC disease modeling and exploit its potential as a new platform for drug discovery and clinical efficacy validation in the near future. Emerging technologies include biomaterial engineering and multiomics. Biomaterials, for example, can increase the compatibility and physiological complexity of the existing cerebral organoids [81]. Poly (lactide-co-glycolide) copolymer (PLGA) fiber microfilaments have been used as a floating scaffold to elongate EBs and retain their self-assembly capacity to form brain organoids. A scaffold-supported SFEBq system can significantly improve the development of neuroectoderm and cortical structures of the derived brain organoids. Reconstituted basement membranes can also enable the spatial organization of the polarized cortical plate and radial units of a cortical structure. Therefore, inclusion of bioengineering can largely improve tissue organization and enhance the recapitulation of physiological complexity of the human brains.

Multiomic studies have been increasingly incorporated in iPSC disease modeling. Human genome project has led a rocketing advancement in biotechnology since initiated in 1990, ranging from genome sequencing to epigenomic, transcriptomic, and proteomic analyses. The fact that iPSC lines harbor a potential risk of chromosomal abnormalities and epigenomic aberrations makes it essential to evaluate iPSCs’ molecular and cellular functions prior to disease modeling or clinical application [208]. Incorporation of the next-generation sequencing in iPSC disease modeling fulfills the need of genome sequencing with high cost-effectiveness and establishes a real-time correlation between the transcriptomic profiling and phenotypic activities, allowing comparison between the sequencing data and the in vitro cytopathies [34,209]. This system benefits the identification of action mechanisms in pathogenesis of CNS diseases that require a long-term developmental process and manifest differentiation-related transcriptional dysregulation. Single-cell RNA sequencing has also been used to characterize cerebral organoids and evaluate their resemblance to the human brains through genome-wide transcriptomic profiling. In addition, whole-genome analyses help uncover novel biomarkers and facilitate the investigation of disease pathologies [1].

Last, artificial intelligence (AI) emerges as a new promise to revolutionize drug development through AI-powered improvements in R&D efficiency [210]. Traditional drug discovery process has four stages: (1) Target identification through cellular and genomic evaluation, proteomic analyses, and bioinformatic predictions, (2) identification of ‘hit’ compound through combinatorial chemistry, HTS, and virtual screening, (3) in vivo tests on toxicity and pharmacokinetics, and (4) clinical trials [211]. In primary screening, AI technology is trained to extract cellular features for target identification. For example, tamura- and wavelet-based texture features are extracted from cell imaging to classify breast cancer cells [212,213]. By implementing this automatic analytic system, it is easier to identify a ‘hit’ compound that presents bioactivity against a specific enzymatic target. Application of iPSCs enables AI-powered HTS on a more physiologically reliable model to elevate the efficiency and accuracy of drug screening. Similarly, structure-activity, in silico analyses and cellular functions are assessed in an iterative process of hit optimization to improve the pharmacological efficacy of drug candidates [211]. AI also automates compound designs by incorporating molecular fingerprint in algorithms [214]. The DeepTox algorithm is one example that utilizes machine learning and history of predefined toxicophore features to predict 12,000 environmental chemicals and drugs with high accuracy [215,216,217]. Accordingly, an AI-powered analytical system promotes the drug discovery cycle by offering more efficient and accurate drug designs to lead a new tide of drug innovation in the short future.

## 8. Conclusions Remark

A constant high attrition rate of neurotherapeutics and low efficacy of clinical translation are derived from mismatch outcomes between disease modeling and human pathophysiology. Development of iPSCs shifts the paradigm of traditional drug development and provides a new opportunity to overcome the existing challenges in disease modeling and in vitro prediction of human physiological responses. An iPSC-based disease modeling is particularly favored in drug discovery due to their scalability for large-scaled, fast, high-throughput screening (HTS), pluripotency to generate multiple independent disease-relevant cell types, patient-specific genetic backgrounds to develop personalized therapy, and flexibility to integrate with multi-disciplinary technologies for extension of the iPSC application. In comparison with 2D human iPSC models, 3D iPSC models exhibit de novo pathological phenotypes overlooked in a 2D culture and provide cytoarchitectural complexity and intercellular cross-talks to facilitate the maturation of cellular disease models with mircroenvironmental signals comparable to those of in vivo pathophysiology. Researchers have recently accomplished in developing a variety of 3D human iPSC models targeting CNS diseases, including BBB models, vascularized cerebral organoids, and microfluidic-based organs-on-chip. All of these models present a great promise to explore disease mechanisms of action and promote drug discovery and modification. Additionally, iPSC models integrated with multi-disciplinary technologies in bioengineering and AI-powered “Omics” approaches have encouraged the development of potent disease models to revolutionize the process of drug development.
